# Risk assessment in deferred and recovered donors

**DOI:** 10.1007/s10561-021-09909-9

**Published:** 2021-03-19

**Authors:** Ellen Heck, Valerie Corder, Jill Urban, Matthew Petroll, William Timmons, Dwight Cavanaugh

**Affiliations:** UT Southwestern Medical Center, Southwestern Institute of Forensic Sciences, Dallas, TX USA

**Keywords:** Tissue Donation: Donor Risk Assessment Interview (DRAI) screening, Serologic testing

## Abstract

Serologic testing and Donor Risk Assessment Interview (DRAI) combined have made tissue transplantation a frequent and safe modality for a variety of trauma and disease conditions. Donate Life America reports 30,000 tissue donors providing more than 1,750,000 tissue transplants annually. This study of 188 potential donor cases addresses issues of risk assessment in a medical examiner population in a metropolitan area, where serologic testing of deferred potential donors were compared with the DRAI screening, which determined the suitability or non-suitability for tissue procurement. Such serologic testing of deferred cases is not usually available in evaluating screening processes. This comparison gives insight into the effectiveness of the DRAI screening in deferring potential serology reactive donors. Results show in 65 cases how the DRAI screening eliminates most, but not all of the serologically reactive donors identified post recovery. The result emphasizes the need for the combined process of DRAI screening and testing to assure transplantation safety.

## Introduction

Serologic testing and the Donor Rick Assessment Interview (DRAI) combined have made tissue transplantation a frequent and safe treatment modality for a variety of trauma and disease conditions. Donate Life America reports 30,000 tissue donors providing more than 1,750,000 tissue transplants annually (Donate Life America Jan 2019).

The Eye Bank Association of America reports over 68,700 ocular donors with approximately 136,100 tissues recovered annually (Eye Bank Association of America 2019). Contraindications and disease transmissions from these 68 thousand transplants are rare to non-existent (Centers for Disease Control and Prevention August 2012). Nevertheless, those involved in the delivery of allografts are always concerned that the screening techniques are appropriately applied, and potential donations are realized or deferred based on the accurate application of the DRAI, and that serology reactive donors are not identified after tissue retrieval. This study addresses these issues in a medical examiner population in a metropolitan area where testing of deferred potential donors could be compared with the DRAI screening which determined the suitability or non-suitability for tissue procurement. This comparison gives some insight into the effectiveness of the screening in deferring potential serology reactive donors.

## Methods

In an eight-month period (July 5, 2019–February 29, 2020), 188 potential donors that were Medical Examiner cases were reviewed. All potential donors, even those deferred after review of Medical Examiner Field Investigator reports, physical assessment by transplant technicians or DRAI, were tested for the standard required FDA (Food and Drug Administration) serologic tests (US Food and Drug Administration 21 CFR 1271 Guidance to Industry). Testing included Ultrio Nucleic acid, NAT, for Hepatitic C and HIV, Hepatitis B antigen and antibody and core Hepatitis C antigen and antibody and syphilis. The results of reactive, nonreactive and risk deferral reasons were compared to evaluate the appropriateness of suitability or deferral of tissue for recovery. Where recovered donors are found to have reactive serologies, these results are not obtained until after recovery and non recovered potential donors are not customarily tested. Data between the two groups, deferred and tested, was subjected to Chi-square analysis.

## Results

**Fig. 1:**
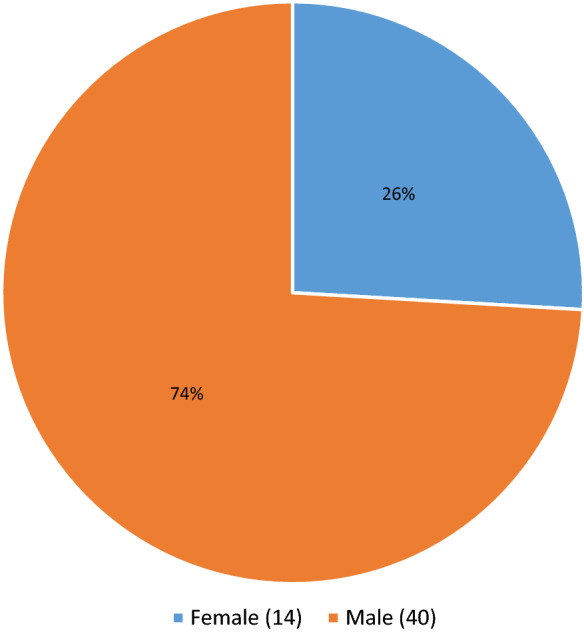
ME case deferrals predominately in male population

**Fig. 2:**
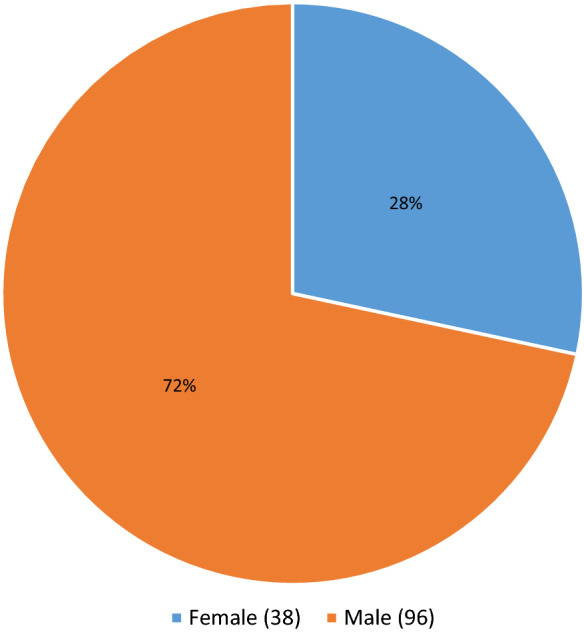
Recovered Case Gender Distribution Predominately Male

**Fig. 3:**
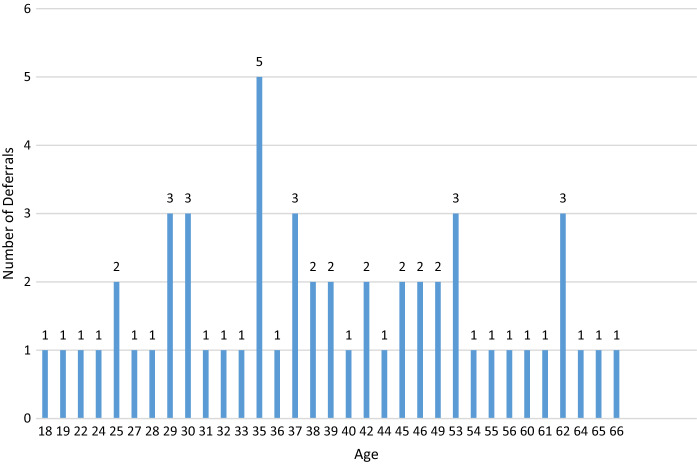
Age range between 35 and 53 years for greatest number of Deferred Medical Examiner cases with overall range between 18 and 66 years

**Fig. 4:**
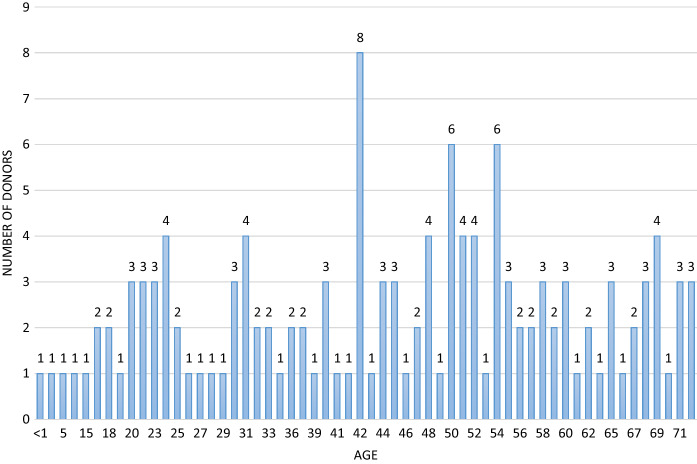
Age range between 4 months and 71 years with greatest concentration between 31 and 54 years

Of the 188 cases, 54 cases were deferred based on incarceration, poly substance abuse, high risk sexual practices and suspected IV drug use, without next of kin contact, or medical social history review with the DRAI. Of these 54 deferred cases, 20 had one or more reactive serologies. The testing results for the deferred Medical Examiner’s cases can be seen in Table 1. 134 cases were recovered as potentially suitable donors with the DRAI, next of kin review, and donor physical assessment. In the 134 recovered donors after the DRAI and physical assessment appeared to indicate no high-risk issues, seven had reactive serologies as seen in Table 2. The comparison of these two populations for ratio of reactive serology was significantly higher for the deferred group Chi-square = 34.543 with one degree of freedom.(*P* =  ≤ 0.001). The male to female ratio in deferred cases was 40 males to 14 females for the deferred cases Fig. 1. In the tissue-recovered group, there were 96 males and 38 females Fig. 2. The male to female ratio showed no significant difference with a Chi-square + 0.0247 and (*P* = 0.874). Age range in the deferred Medical examiner cases was 18 years to 66 years Fig. 3. The tissue- recovered group ranged in age from 4 months to 72 years Fig. 4. In the tissue- recovered population, all seven of the reactive serologies were from female donors (Table 2). One history obtained by the organ procurement agency, was from an individual with a history of bipolar depression, anxiety and cocaine use at least 15 years prior. Her serology was reactive for Hepatitis C. One other case, also an organ procurement agency screened case, was reactive for HIV1/2 UltroHIV-1 (Human Immunodeficiency Virus). Corneas were the only tissue recovered, but were not transplanted. There were no identified risk factors at the time of recovery, as defined in 21 CFR 1271 of FDA Guidance document, to require deferral. Autopsy indicated hepatic cirrhosis and upper gastric intestinal bleed. The initial Medical Examiner’s Report indicated cirrhosis and the DRAI indicated a past history of heroin use. The heroin use was reported to have been “a couple of times a week approximately 10–15 years ago” and not since 2006. It was also reported that alcohol was not consumed since 2013. The initial Medical Examiner’s Report did not indicate any concern other than the hepatic cirrhosis. This initial Medical Examiner report is a gross observation by the pathologist of external and internal inspection and any history known by the Medical Examiner’s office and does not include any histology or serology. Of the remaining donors, four tissue recovery cases were Hepatitis B serology reactive. Two of these cases were Hepatitis B core reactive, and two were Hepatitis B antigen reactive. There was one RPR, Rapid Plasma Reagent case recovered. A confirmatory test for syphilis is permitted by FDA Guidance (US Food and Drug Administration 21 CFR 1271 Guidance to Industry), but is not available in this case. There was also one HIV reactive serology in the deferred group from a 56 year old male who was found with in a hotel room with a used needle and other drug related items. Only two reactive Ultrio HIV 1 reactive serologies were present in the total 188 potential donor cases. There were four hepatitis B cases and two were antigen positive. A 67 year old female who was antigen reactive had no medical history except for gastric ulcers and gastric hemorrhage. She had lived in India for 65 years and was only in The United States for the last two and a half years. Autopsy findings indicated cirrhosis. The second antigen reactive donor was a 55 year old female with a history of hypertension and heart disease and no high risk indications. For the Hepatitis B antibody or core reactive cases, one had a history of alcohol abuse and some use of marijuana and cocaine, which was not reported at the initial screening. The second had a history of dialysis for renal disease, hypertension and prior myocardial infarction, with no high risk factors. A 51 year old female who died of hypertensive cardiovascular disease had a past history of cocaine and marijuana use 15–20 years earlier, and was Hepatitis C reactive for both NAT and antibody. The one syphilis case, which may or may not have been a true reactive, since no confirmatory was available, had no identified risk and had a medical history of hypertension and congestive heart disease. No serology reactive donor tissue was transplanted.

**Table 1:**
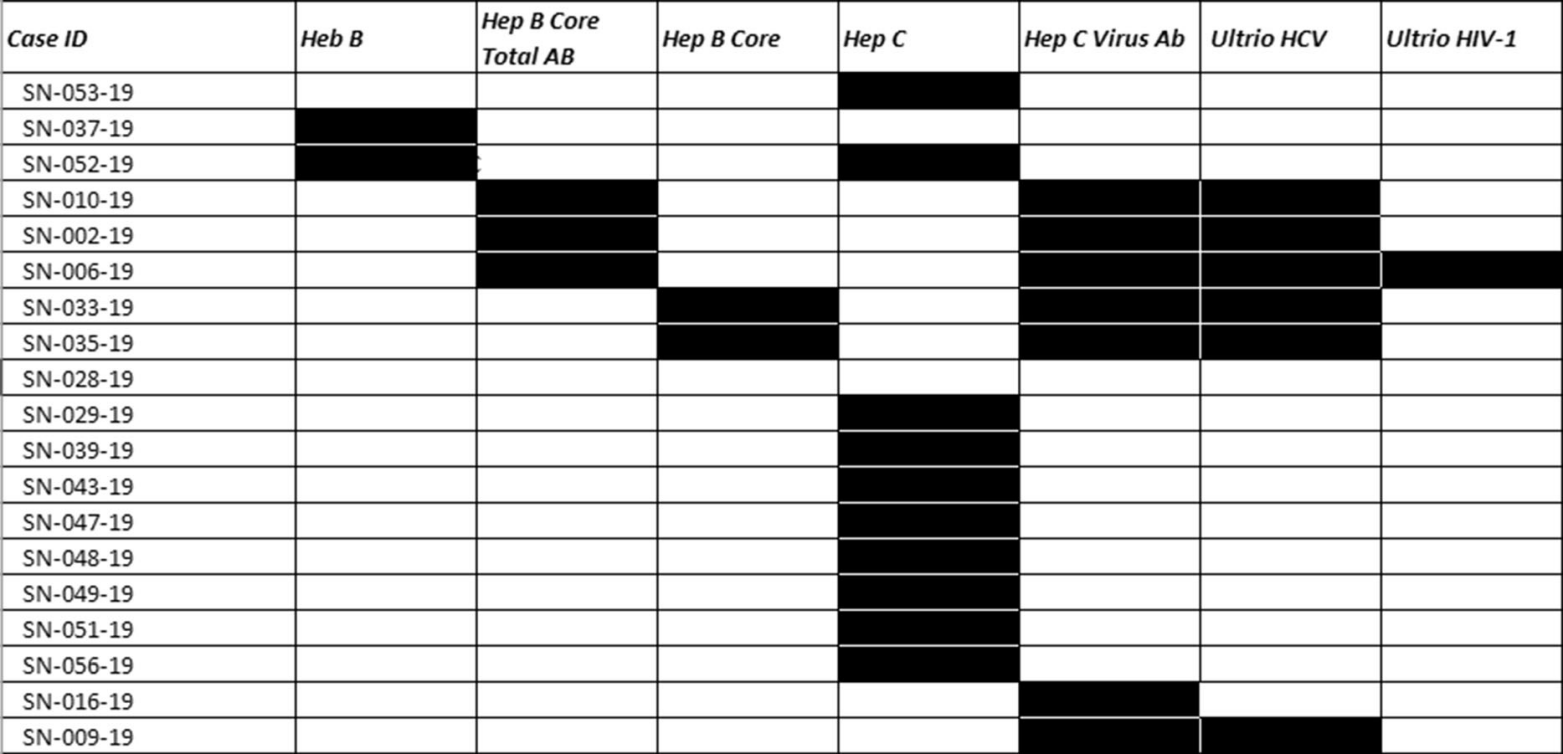
Deferred medical examiner cases with reactive serology

Hepatitis C virus demonstrated highest number of reactive tests in this deferred population

**Table 2:**
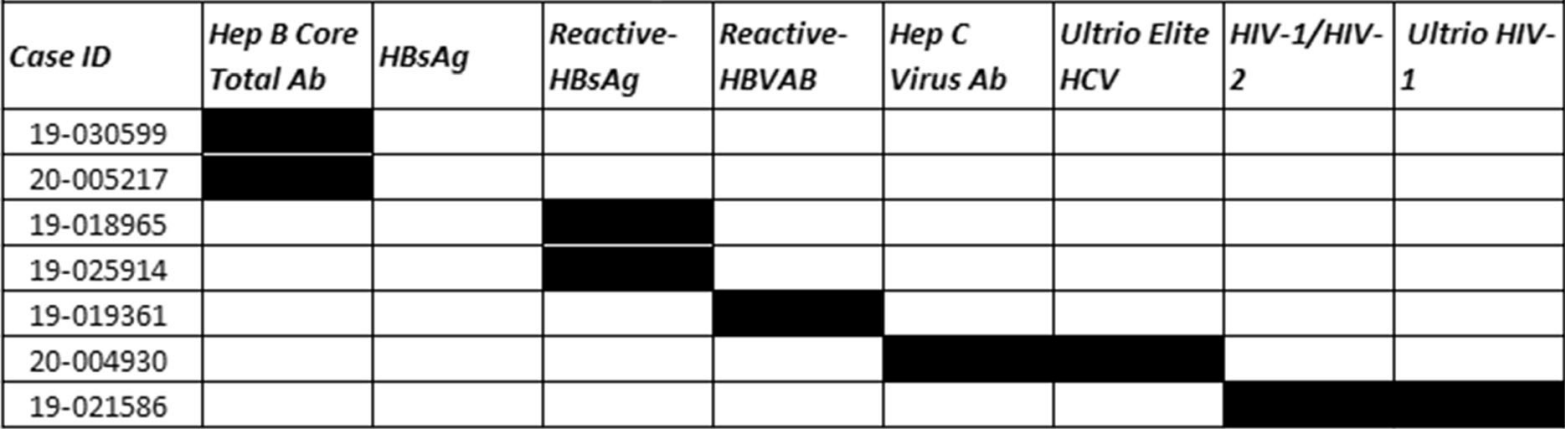
DRAI screened donors with reactive serology

Prescreened donors reactive serology identified after tissue recovery. Most prevalent for Hepatitis B in 5 of the 7 cases

## Discussion

The testing results from cases that have been deferred at initial history screening are not customarily available due to limitations in access to the body; expense and time involved sending a technician to draw blood; testing costs, and the need to release the body to the funeral home in a timely manner. In these Medical Examiner cases, the testing was available because of a supportive Medical Examiner’s office, access to the case under the jurisdiction of the Medical Examiner and an interest in evaluation of the effectiveness of the screening process with the subsequent testing results. In addition, a major national reference laboratory donated the cost of testing. This unique combination of circumstances allowed for data not previously accessible and of significant value in demonstrating the risk factors for allograft tissues and the value of the current screening and testing practices. The data shows the continued interdependence and effectiveness of the combination of donor screening and serology testing in providing for the safety of donor allograft tissues for transplantation. Hepatitis C was the most frequently reactive serology in the deferred cases. This serology reactivity was correlated most often with a history of IVD use (intravenous drug use), and was significantly less frequent with the history of poly substance abuse (non- IV drugs, prescription meds, recreational). The numbers for incarceration were too limited to be significant, but were also reactive for Hepatitis C (Table 3).  The single donor case where the serology was reactive for HIV had no current identifiable history to have supported concern for a reactive serology finding. The donor, a 57-year-old female, had a current history of a gastro-intestinal bleed and hepatic cirrhosis. She had been a heroin user “2 to 3 times a week, 10 to 15 years prior”. The historian in this case was the son and he was apparently unware of any current drug usage. The case was re-reviewed to look for any additional information that might have been overlooked in the original assessment, but none was found. The association of the prior drug use and the reactive HIV serology with no information on any treatment history for AIDS is perplexing. All seven of the reactive serology donor cases were re-examined for any possible risk history that might have caused deferral based on history alone. No history was found that was not acceptable by the FDA screening criteria. However, a more liberal interpretation might have suggested two of the cases with drug use greater than 10 years prior was the explanation or contributor for their reactive serologies. In these cases, a more remote use of drugs as the causative agent for the Hepatitis B finding might suggest a chronic asymptomatic hepatitis resulting in the reactive hepatitis serology. There is no correlation of cirrhosis and HIV other than the possible damage of the drugs to the liver (Kaspar and Sterling October 2017). In the Medical Examiner cases the tissue bank deferred, the serologies were reactive in 11 cases where IVD use was reported or suspected, which was the area of highest occurrence in these “high risk” cases. However, there were the same number of non-reactive serology tests in “high risk” IVD or suspected IVD use. Practices of shared needle usage or indiscriminate sexual practice more commonly associated with HIV (Gonzalez Gompf. HIV Infection Facts, History and Causes), were not contained in the history reviews and may or may not have been known to the historian in the recovered cases. Serology testing and reduction of the window period between infection and antigen or antibody production has been reduced but does still exist (National Institute of Allergy and Infectious Diseases May 2019), and therefore cannot be the sole determining factor for donor suitability. Even a 12-day window period could be the difference between safety and risk for donated tissue in some circumstances. Window period refers to the time in which viral levels may not be detected even with the highly sensitive nucleic acid test, a period of 10 to 12 days, but may be as long as 33 days. The cases deferred due to IVD or suspected IVD use which were non-reactive might be within the window period. They could also have been on medication reducing their detectability, or be true non-reactive and still be within a group where high risk issues must be suspected. This is concerning where sterilization of other processing treatment does not remove the cellular components of the graft, (Science Direct. Terminal Sterilization 2019. Parsons –Sterilisation Procedures for Tissue Allografts 2013). In tissue donation the historian is, unlike in blood donation, a second hand source and therefore always in question as to the depth and accuracy of information (AOPO-EBAA-AATB Guidance Document). However, based on these cases, it would appear trained interviewers using the provided screening tool, the DRAI, are accessing the donor risk status with significant accuracy.

**Table 3:** Deferred donors—Reactive serology

High risk (54 total)	Non-reactive	Recative	Total
IVDA	11	11	22
Incarceration	7	3	10
Polysubstance abuse	12	4	16
Sexual high risk	4	2	6
Totals	34	20	54

## Conclusion

Reactivity of communicable disease serologic markers as defined by the FDA in 21 CFR 1271, appears to be most clearly associated with intravenous drug use. Other risk indicators i.e. incarceration, substance abuse, non-IV drugs, and sexual activity were insufficient numbers to establish any significant conclusions. What is however apparent from these 188 cases is the significant role of the medical social history screening, DRAI, and serology results in assuring the safety of donor tissues for transplantation. Therefore, the selection of a reference laboratory familiar with the testing requirements for cadaveric samples using the most sensitive and specific testing modalities, and a well-trained screening staff conducting donor family risk assessment interviews is essential to the safety of allograft transplantation healthcare delivery.

No ethical rules or conflict of interest rules were violated/Dataset generated/used in the current study is available at UT Southwestern Medical Center.
